# Squamous cell carcinoma predicts worse prognosis than adenocarcinoma in stage IA lung cancer patients: A population-based propensity score matching analysis

**DOI:** 10.3389/fsurg.2022.944032

**Published:** 2022-08-23

**Authors:** Bo Hao, Fang Li, Xiaoxia Wan, Shize Pan, Donghang Li, Congkuan Song, Ning Li, Qing Geng

**Affiliations:** ^1^ Department of Thoracic Surgery, Renmin Hospital of Wuhan University, Wuhan, China; ^2^ Department of Ophthalmology, The First Hospital of Wuhan, Wuhan, China; ^3^ Department of Cardiothoracic Surgery, Ezhou Central Hospital, Ezhou, China

**Keywords:** lung squamous cell carcinoma, lung adenocarcinoma, histological type, overall survival, lung cancer–specific survival

## Abstract

**Background:**

Although numerous studies have reported the association between histological types and the prognosis of IA non-small-cell lung cancer (NSCLC) patients, few studies have deeply investigated the impact of pathology on the outcome of NSCLC patients. In this study, we comprehensively explored whether the type of histology influenced the outcome of IA-stage NSCLC patients.

**Methods:**

The study population was obtained from the Surveillance, Epidemiology, and End Results (SEER) program, which is supported by the National Cancer Institute of the United States. To avoid potential bias, the method of propensity score matching (PSM) was used to obtain a balanced cohort for further analysis.

**Results:**

The results from univariate and multivariate regression models showed that lung squamous cell carcinoma (LSQCC) patients were at a significantly greater risk of undergoing shorter overall survival (OS) and lung cancer–specific survival (LCSS). After PSM analysis, LSQCC was still closely associated with a reduction in OS and LCSS. All of these suggested that the histological type was an independent prognostic factor for OS and LCSS.

**Conclusion:**

Our study demonstrated that squamous cell carcinoma predicted worse OS and LCSS in IA-stage NSCLC patients compared with lung adenocarcinoma (LUAD). We suggest that the outcomes of LSQCC and LUAD are very different and that the two histological types should be differently analyzed.

## Introduction

Lung cancer is the leading cause of cancer-related death and thus remains a major public health problem worldwide (1). Non-small-cell lung cancer (NSCLC) accounts for 85% of all cases. Although lung adenocarcinoma (LUAD) has replaced lung squamous cell carcinoma (LSQCC) as the most common case of NSCLC, there is still a large number of patients who are diagnosed with LSQCC (2). A growing number of researchers have come to realize that there are significant differences in prognostic factors and outcomes between LUAD and LSQCC (3–7). Although numerous studies have investigated the relationship between histological types and survival, few studies have comprehensively explored the impact of histological types on survival.

As a result of the difference in the outcomes of adenocarcinoma and squamous cell carcinoma, the Cancer Staging Manuals based on the American Joint Committee on Cancer (AJCC) for esophageal adenocarcinoma and squamous cell carcinoma began to be separated since the 7th edition. A similar scenario was also observed in NSCLC. Studies have shown that the prognosis of LSQCC is better than that of LUAD (3, 4), but other studies have shown that the prognosis of LSQCC is worse (5–7). Therefore, the prognostic role of histological types in NSCLC needs to be further studied.

In this study, subjects were obtained from the Surveillance, Epidemiology, and End Results (SEER) database between 2004 and 2013 to investigate the impact of the histological types on survival in patients with IA-stage NSCLC.

## Materials and methods

### Data source

The study population in our study is from the SEER project, which is funded by the National Cancer Institute of the United States. The project provides clinical characteristics (such as histology, tumor size, tumor, primary tissue location, TNM stage, etc.), treatment strategies (lobectomy and sublobar resection), and survival time, which facilitate researchers to study the prognostic significance of clinical characteristics. Many high-quality studies have been published using data from the SEER database (8–11).

### Inclusion criteria

The selection criteria for this study are as follows: (i) pathologically confirmed primary non-small cell lung cancer, and only squamous cell carcinoma and adenocarcinoma; (ii) tumor size less than 3 cm, and no lymph nodes or distant organ metastasis; (iii) excluding no surgery history or patients who received extended lobectomy, total lobectomy, or extended lobectomy; (iv) excluding patients who received chemotherapy before or after surgery; (v) excluding patients who received radiotherapy treatment before or after surgery; (vi) patients >18 years old; (vii) excluding patients with the tumor located in the main bronchus; and (viii) excluding patients with survival time ≤6 months.

### Data collection

The study population was obtained from the SEER database between 2004 and 2014. The baseline items in this study included demographic characteristics (age, gender, and race), tumor features (histological type, differentiation grade, tumor size, location, laterality, and TNM stage), and treatment strategies (lobectomy and sublobar resection). Histological type identification: 8140–8147, 8244, 8245, 8250–8255, 8260, 8290, 8310, 8320, 8320, 8323, 8330–8332, 8470, 8480–8481, 8550–8551, and 8570–8573 were identified as adenocarcinoma, and 8052, 8070–8075, 8078, and 8083–8084 were identified as squamous cell carcinoma (12–14). Surgical procedures (SPs) were divided into sublobar resection (SR) (code: 21, 22) and lobectomy (LT) (code: 30, 33). SR included wedge resection and segmentectomy.

The primary endpoints in our study were overall survival (OS) and lung cancer–specific survival (LCSS). OS was identified as the time from diagnosis to death, and LCSS was defined as the time from diagnosis to death due to lung cancer. Patients who were still alive at the end of the follow-up or who died from other causes were defined as censored patients.

### Statistical analysis

The Wilcoxon test was performed to calculate the difference in the distributions of continuous data (such as age, number of resected regional lymph nodes, and TS), while the Pearson *χ*^2^ test was used to assess the difference in categorical variables (such as gender, location, laterality, histological type, and differentiation grade). The Kaplan–Meier method was used to establish the curves of OS and LCSS, and the differences were calculated by using a log-rank test. Survival comparisons of all prognostic factors were analyzed by performing univariate Cox regression analysis, and multivariate analysis was performed only when univariate analysis indicated that there was a statistical significance. To balance clinical variables and reduce potential selection bias, propensity score matching (PSM) was used (15, 16). Due to the great heterogeneity of variables, a one-to-one nearest-neighbor matching with the caliper set at 0.001 was used to create a balanced cohort.

All *P* values in our study were two-sided, and only when *P* < 0.05 was considered to indicate a statistical significance. In univariate and multivariate Cox regression models, the hazard ratios (HRs) and their 95% CIs for survival analyses were calculated using SPSS 22.0 (IBM, Armonk, NY). Propensity score-matched analysis and survival curves were established by R 4.0.1 (R, Vienna, Austria).

## Results

### Baseline characteristics of the population

After selection, a total of 27,398 patients with T1N0M0 NSCLC (only adenocarcinoma and squamous cell carcinoma) were included (detailed strategies shown in Supplementary Figure S1), of whom 20,499 patients were pathologically confirmed LUAD and 6,899 LSQCC. Among them, 11,794 were male and 15,604 were female. The study population spanned from 1 January 2004 to 31 December 2013. The mean age for the study population was 68.47 (±9.37) years. The median follow-up time for the LUAD patients was 65 months and for LSQCC patients was 57 months. The detailed descriptions of the subgroups of each variable and the correlation between each variable and histology are presented in Table 1. Compared with patients diagnosed with adenocarcinoma, LSQCC patients were more likely to be elderly and male. Additionally, the squamous cell carcinoma seems more likely to be located in the left lung and has a poor tumor differentiation grade compared with LUAD patients. After PSM analyses, 6,423 pairs were created in the adenocarcinoma and squamous cell carcinoma groups. There was no significant difference in any subgroup. In addition, the distributions of the propensity score were balanced between the two groups (Supplementary Figure S2 and Table 1).

**Table 1:** Baseline characteristics before and after matching.

	Full cohort			Matched cohort		
	SCC (*n *= 6,899)	Adenocarcinoma (*n *= 20,499)	*P* value	SCC (*n *= 6,423)	Adenocarcinoma (*n *= 6,423)	*P* value
Age			<0.001*			0.386
≤69	3,064 (44.4%)	11,078 (54.0%)		2,948 (45.9%)	2,997 (46.7%)	
>69	3,835 (55.6%)	9,421 (46.0%)		3,475 (54.1%)	3,426 (53.3%)	
Gender			<0.001*			0.958
Male	3,706 (53.7%)	8,088 (39.5%)		3,408 (53.6%)	3,405 (53.0%)	
Female	3,193 (46.3%)	12,411 (60.5%)		3,015 (46.4%)	3,018 (47.0%)	
Race			<0.001*			0.952
Caucasian	6,192 (89.8%)	17,508 (85.4%)		5,808 (91.2%)	5,810 (90.4%)	
Others	707 (10.2%)	2,991 (14.6%)		615 (8.8%)	613 (9.6%)	
Grade			<0.001*			0.056
Well/moderate	3,786 (54.9%)	15,304 (74.7%)		3,747 (60.4%)	3,754 (60.9%)	
Poor/UD	2,843 (41.2%)	3,709 (18.1%)		2,434 (36.1%)	2,375 (35.3%)	
Unknown	270 (3.9%)	1,486 (7.2%)		242 (3.5%)	294 (38%)	
Resected LNs			0.001*			0.926
0	1,095 (15.9%)	2,873 (14.0%)		970 (15.1%)	979 (15.2%)	
1–3	1,373 (19.9%)	3,979 (19.4%)		1,266 (19.7%)	1,260 (19.6%)	
≥4	4,078 (59.1%)	12,655 (61.7%)		3,894 (60.6.5%)	3,906 (60.8%)	
Unknown	353 (5.1%)	992 (4.8%)		293 (4.7%)	278 (4.3%)	
Tumor size (mm)			<0.001*			0.921
≤10	755 (10.9%)	2,924 (14.3%)		689 (10.7%)	684 (10.6%)	
11–20	3,505 (50.8%)	11,031 (53.8%)		3,332 (51.9%)	3,332 (52.2%)	
21–30	2,639 (38.3%)	6,544 (31.9%)		2,402 (37.4%)	2,384 (37.1%)	
Surgical procedure			<0.001*			0.981
Sublobar resection	2,037 (29.5%)	5,549 (27.1%)		1,819 (28.3%)	1,848 (28.8%)	
Lobectomy	4,862 (70.5%)	14,950 (72.9%)		4,604 (71.7%)	7,575 (71.2%)	
Location			0.001*			0.859
Upper	4,314 (62.5%)	12,617 (61.5%)		4,125 (64.2%)	4,169 (64.9%)	
Middle	304 (4.4%)	1,134 (5.5%)		268 (4.2%)	268 (4.2%)	
Lower	2,244 (32.2%)	6,530 (31.9%)		1,995 (31.1%)	1,950 (30.4%)	
Others	57 (0.9%)	218 (1.1%)		35 (0.5%)	36 (0.5%)	
Laterality			<0.001*			0.763
Left	3,063 (44.4%)	8,294 (40.5%)		2,860 (44.5%)	2,843 (44.3%)	
Right	3,836 (55.6%)	12,205 (59.5%)		3,563 (55.5%)	3,580 (55.7%)	

UD, undifferentiated; LN, lymph node; SCC, squamous cell carcinoma; *Difference was statistically significant.

### Survival analysis before matching

**Figure 1:**
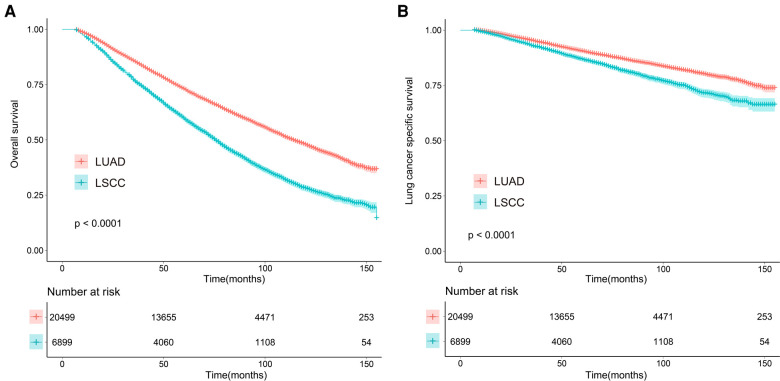
Overall (**A**) and lung cancer–specific survival (**B**) for lung cancer patients with adenocarcinoma and squamous cell carcinoma before matching. LUAD, lung adenocarcinoma; LSQCC, lung squamous cell carcinoma.

As shown in Figure 1A, survival curves established by Kaplan–Meier revealed that patients who were diagnosed with adenocarcinoma had better OS than those diagnosed with squamous cell carcinoma. The results from univariate [hazard ratio (HR) = 1.670, 95% CI [1.608, 1.735], *P* < 0.001] and multivariate (HR = 1.402, 95% CI [1.347, 1.459], *P* < 0.001) regression analyses showed that NSCLC patients with squamous cell carcinoma experienced shorter OS compared with patients with adenocarcinoma (Table 2). As depicted in Figure 1B, LSQCC was associated with poorer LCSS compared with LUAD. The results from multivariate regression analysis indicated that histological type was an independent prognostic factor for LCSS, and LSQCC predicted worse LCSS compared with LUAD (HR = 1.301, 95% CI [1.212, 1.397], *P* < 0.001). As presented in Table 2, we could also find that older age, male, poorer tumor differentiation, a smaller number of resected lymph nodes, and larger tumor size were associated with worse OS and LCSS, respectively. Moreover, NSCLC patients with IA stage who underwent lobectomy had favorable OS (univariate: HR = 0.615, 95% CI [0.592, 0.639], *P* < 0.001; multivariate: HR = 0.763, 95% CI [0.727, 0.800], *P* < 0.001); however, as for LCSS, univariate analysis showed a significant correlation (HR = 0.860, 95% CI [0.798, 0.926], *P* < 0.001), and multivariate analysis showed no significant correlation (HR = 0.934, 95% CI [0.852, 1.023], *P* = 0.141).

**Table 2:** Univariate and multivariate regression analyses for overall survival and lung cancer–specific survival before matching.

	Overall survival						Lung cancer–specific survival					
	HR^a^	95% CI^a^	*P* ^a^	HR^b^	95% CI^b^	*P* ^b^	HR^a^	95% CI^a^	*P* ^a^	HR^b^	95% CI^b^	*P* ^b^
Age												
≤69	1			1			1			1		
>69	1.772	1.708–1.837	<0.001^c^	1.625	1.566–1.687	<0.001^c^	1.226	1.148–1.310	<0.001^c^	1.157	1.082–1.237	<0.001^c^
Gender												
Male	1				1		1			1		
Female	0.687	0.662–0.712	<0.001^c^	0.734	0.708–0.761	<0.001^c^	0.764	0.715–0.816	<0.001^c^	0.821	0.768–0.878	<0.001^c^
Race												
Caucasian	1			1			1					
Others	0.845	0.798–0.893	<0.001^c^	0.907	0.857–0.9601	0.001^c^	0.957	0.867–1.056	382			
Location												
Upper	1						1					
Middle	1.022	0.943–1.108	0.588				0.975	0.839–1.133	0.742			
Lower	1.031	0.992–1.072	0.123				0.981	0.913–1.055	0.607			
Others	1.091	0.916–1.300	0.330				1.197	0.882–1.625	0.248			
Laterality												
Left	1											
Right	0.970	0.935–1.006	0.135				1.014	0.948–1.076	0.754			
Grade												
Well/moderate	1			1			1			1		
Poor/UD	1.429	1.373–1.4787	<0.001^c^	1.267	1.215–1.321	<0.001^c^	1.635	1.522–1.756	<0.001^c^	1.487	1.380–1.602	<0.001^c^
Unknown	0.847	0.993–1.070	0.847	0.993	0.922–1.071	0.864	0.957	0.830–1.104	0.548	0.986	0.855–1.137	0.846
Resected LNs												
0	1			1			1			1		
1–3	0.665	0.629–0.703	<0.001^c^	0.765	0.720–0.812	<0.001^c^	0.874	0.783–0.976	0.016^c^	0.838	0.744–0.944	0.004^c^
≥4	0.503	0.480–0.528	<0.001^c^	0.616	0.581–0.653	<0.001^c^	0.709	0.645–0.780	<0.001^c^	0.680	0.606–0.763	<0.001^c^
Unknown	0.606	0.555–0.662	<0.001^c^	0.708	0.646–0.777	<0.001^c^	0.804	0.681–0.949	0.010^c^	0.773	0.649–0.920	0.004^c^
Tumor size (mm)												
≤10	1						1			1		
11–2	1.065	1.006–1.128	0.029^c^	1.111	1.049–1.178	<0.001^c^	1.410	1.251–1.589	<0.001^c^	1.423	1.261–1.606	<0.001^c^
21–30	1.250	1.178–1.326	<0.001^c^	1.320	1.241–1.403	<0.001^c^	1.936	1.714–2.186	<0.001^c^	1.931	1.704–2.188	<0.001^c^
SP												
SR	1						1			1		
LR	0.615	0.592–0.639	<0.001^c^	0.763	0.727–0.800	<0.001^c^	0.860	0.798–0.926	<0.001^c^	0.934	0.852–1.023	0.141
Histology												
AD	1			1								
SCC	1.670	1.608–1.735	<0.001^c^	1.402	1.347–1.459	<0.001^c^	1.447	1.347–1.555	<0.001^c^	1.203	1.115–1.298	<0.001^c^

HR, hazard ratio; UD, undifferentiated; LN, lymph node; AD, adenocarcinoma; SCC, squamous cell carcinoma; SR, sublobar resection; LR, lobectomy; SP, surgical procedure.

^a^ Univariate analysis.

^b^ Multivariate analysis.

^c^ Difference was statistically significant.

### Survival analysis after matching

**Figure 2:**
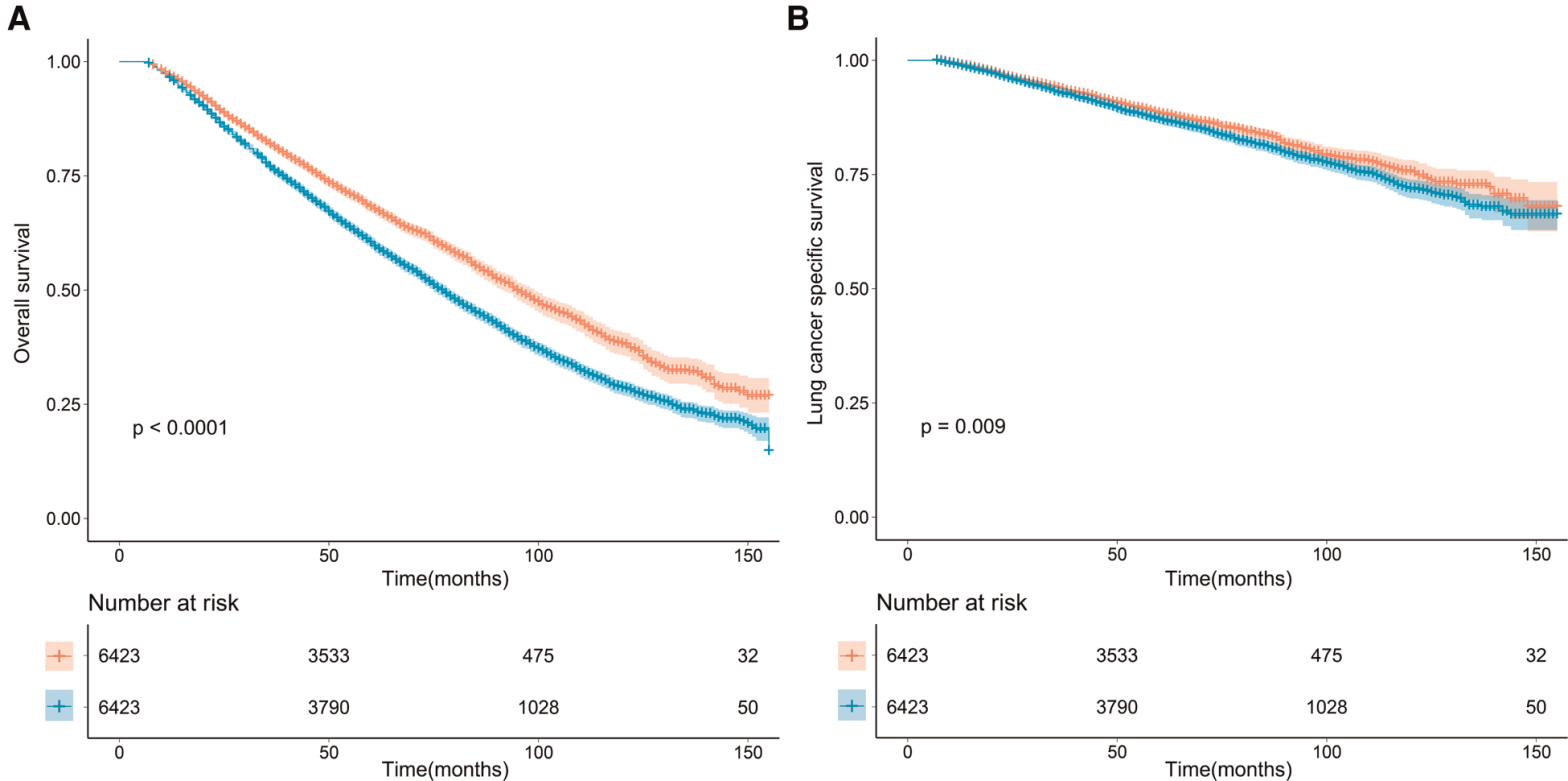
Overall (**A**) and lung cancer–specific survival (**B**) for lung cancer patients with adenocarcinoma and squamous cell carcinoma after matching. LUAD, lung adenocarcinoma; LSQCC, lung squamous cell carcinoma.

To reduce potential selection bias, PSM analysis was conducted. In the balanced cohort, the evidence from survival curves suggested that LSQCC predicted worse OS and LCSS compared with LUAD (shown in Figures 2A,B). In the univariate and multivariate Cox regression models, the results showed that LSQCC patients underwent shorter OS (univariate: HR = 1.310, 95% CI [1.244, 1.381], *P* < 0.001; multivariate: HR = 1.339, 95% CI [1.271, 1.411], *P* < 0.001) and LCSS (univariate: HR = 1.137, 95% CI [1.032, 1.252], *P* = 0.009; multivariate: HR = 1.160, 95% CI [1.053, 1.277], *P* = 0.003) compared with LUAD (Table 3). Patients with lobectomy experienced favorable OS but not LCSS compared with those who received sublobar resection. In addition, age, gender, tumor differentiation grade, the number of resected lymph nodes, and tumor size were independent prognostic factors for OS and LCSS, as indicated in multivariate Cox regression analyses (Table 3).

**Table 3:** Univariate and multivariate regression analyses for overall survival and lung cancer–specific survival after matching.

	Overall survival						Lung cancer–specific survival					
	HR^a^	95% CI^a^	*P* ^a^	HR^b^	95% CI^b^	*P* ^b^	HR^a^	95% CI^a^	*P* ^a^	HR^b^	95% CI^b^	*P* ^b^
Age												
≤69	1			1			1			1		
>69	1.561	1.481–1.644	<0.001^c^	1.515	1.437–1.597	<0.001^c^	1.118	1.016–1.230	0.022^c^	1.119	1.016–1.231	0.022^c^
Gender												
Male	1			1			1			1		
Female	0.802	0.761–0.844	<0.001^c^	0.780	0.741–0.821	<0.001^c^	0.883	0.802–0.971	0.010^c^	0.890	0.808–0.979	0.016^c^
Race												
Caucasian	1			1			1					
Others	0.866	0.791–0.950	0.002^c^	0.892	0.813–0.977	0.011^c^	1.031	0.879–1.209	0.705			
Location												
Upper	1						1					
Middle	1.055	0.931–1.196	0.400				1.195	0.959–1.490	0.113			
Lower	1.039	0.983–1.099	0.177				1.004	0.904–1.115	0.946			
Others	0.865	0.611–1.225	0.413				1.040	0.574–1.884	0.896			
Laterality												
Left	1						1			1		
Right	1.010	0.960–1.063	0.706				1.108	1.007–1.220	0.036^c^	1.118	1.015–1.230	0.023^c^
Grade												
Well/moderate	1			1			1			1		
Poor/UD	1.134	1.076–1.195	<0.001^c^	1.189	1.128–1.253	<0.001^c^	1.390	1.261–1.532	<0.001^c^	1.383	1.254–1.525	<0.001^c^
Unknown	1.023	0.900–1.162	0.730	1.081	0.951–1.229	0.234	1.070	0.837–1.368	0.588	1.125	0.880–1.440	0.348
Resected LNs												
0	1			1			1			1		
1–3	0.680	0.628–0.736	<0.001^c^	0.778	0.713–0.849	<0.001^c^	0.941	0. 801–1.106	0.463	0.855	0.726–1.007	0.060
≥4	0.555	0.519–0.593	<0.001^c^	0.666	0.612–0.726	<0.001^c^	0.829	0.722–0.953	0.008^c^	0.731	0.634–0.842	<0.001^c^
Unknow*n*	0.621	0.545–0.707	<0.001^c^	0.751	0.654–0.861	<0.001^c^	0.823	0.638–1.062	0.134^c^	0.760	0.588–0.982	0.036^c^
Tumor size (mm)												
≤10	1			1			1			1		
11–20	1.041	0.954–1.136	0.365	1.098	1.005–1.188	0.039^c^	1.280	1.063–1.541	0.009^c^	1.280	1.062–1.543	0.009^c^
21–30	1.139	1.042–1.246	0.004^c^	1.290	1.176–1.415	<0.001^c^	1.755	1.457–2.113	<0.001^c^	1.775	1.469–2.145	<0.001^c^
SP												
SR	1			1			1					
LT	0.644	0.610–0.680	<0.001^c^	0.759	0.708–0.813	<0.001^c^	0.920	0.827–1.025	0.130			
Pathology												
AD	1			1			1			1		
SCC	1.310	1.244–1.381	<0.001^c^	1.339	1.271–1.411	<0.001^c^	1.137	1.032–1.252	0.009^c^	1.160	1.053–1.277	0.003^c^

HR, hazard ratio; UD, undifferentiated; LN, lymph node; AD, adenocarcinoma; SCC, squamous cell carcinoma; SR, sublobar resection; LR, lobectomy; SP, surgical procedure.

^a^ Univariate analysis.

^b^ Multivariate analysis.

^c^ Difference was statistically significant.

### Subgroup analyses of the population stratified by tumor size

The prognosis of early-stage NSCLC patients was closely associated with tumor size. Thus, the full cohort was divided into three subgroups according to the tumor size. Because multivariate regression analysis was more reliable than univariate analysis, only multivariate analyses were shown. In Table 4, the results show that adenocarcinoma yielded better OS compared with squamous cell carcinoma in different tumor sizes. Similar results were also observed in LCSS (Table 5). Moreover, gender, the number of resected lymph nodes, and tumor differentiation grade were independent prognostic factors for OS and LCSS regardless of the tumor size (Tables 4 and 5).

**Table 4:** Multivariate regression analyses for overall survival stratified by tumor size.

	Tumor size ≤ 10 mm			10 mm < tumor size ≤ 20 mm			20 mm < tumor size ≤ 30 mm		
	HR	95% CI	*P*	HR	95% CI	*P*	HR	95% CI	*P*
Age									
≤69	1			1			1		
>69	1.582	1.426–1.756	<0.001^a^	1.677	1.592–1.766	<0.001^a^	1.569	1.475–1.668	<0.001^a^
Gender									
Male	1			1					
Female	0.722	0.651–0.801	<0.001^a^	0.926	0.691–0.764	<0.001^a^	0.747	0.703–0.793	<0.001^a^
Grade									
Well/moderate	1			1			1		
Poor/UD	1.267	1.114–1.441	<0.001^a^	1.311	1.236–1.389	<0.001^a^	1.221	1.142–1.306	<0.001^a^
Unknown	1.013	0.855–1.200	0.865	0.977	0.879–1.086	0.667	1.016	0.887–1.164	0.819
Resected LNs									
0	1			1			1		
1–3	0.875	0.753–1.017	0.081^a^	0.777	0.716–0.843	<0.001^a^	0.683	0.620–0.776	<0.001^a^
≥4	0.645	0.555–0.750	<0.001^a^	0.631	0.583–0.683	<0.001^a^	0.573	0.514–0.638	<0.001^a^
Unknown	0.724	0.553–0.947	0.018^a^	0.661	0.581–0.751	<0.001^a^	0.747	0.638–0.875	<0.001^a^
SP									
SR	1			1			1		
LT	0.796	0.699–0.907	0.001^a^	0.743	0.697–0.792	<0.001^a^	0.780	0.716–0.849	<0.001^a^
Pathology									
AD	1			1			1		
SCC	1.489	1.322–1.678	<0.001^a^	1.421	1.343–1.503	<0.001^a^	1.355	1.269–1.447	<0.001^a^

UD, undifferentiated; LN, lymph node; AD, adenocarcinoma SCC, squamous cell carcinoma; SR, sublobar resection; LR, lobectomy; SP, surgical procedure.

^a^ Difference was statistically significant.

**Table 5:** Multivariate regression analyses for lung cancer–specific survival stratified by tumor size.

	Tumor size ≤ 10 mm			10 mm < Tumor size ≤20 mm			20 mm <Tumor size ≤30 mm		
	HR	95% CI	*P*	HR	95% CI	*P*	HR	95% CI	*P*
Age									
≤69				1			1		
>69				1.170	1.064–1.286	0.001^a^	1.151	1.038–1.276	0.008^a^
Gender									
Male	1			1					
Female	0.750	0.599–0.938	0.012^a^	0.790	0.719–0.868	<0.001^a^	0.879	0.792–0.975	0.015^a^
Grade									
Well/moderate	1			1			1		
Poor/UD	1.550	1.192–2.016	0.001^a^	1.548	1.392–1.722	<0.001^a^	1.417	1.265–1.588	<0.001^a^
Unknown	0.846	0.569–1.257	0.407	0.964	0.786–1.183	0.726	1.086	0.862–1.369	0.482
Resected LNs									
0				1			1		
1–3				0.837	0.712–0.983	0.030^a^	0.798	0.650–0.981	0.032^a^
≥4				0.662	0.566–0.774	<0.001^a^	0.660	0.542–0.804	<0.001^a^
Unknown				0.686	0.536–0.879	0.003^a^	0.813	0.613–1.079	0.152
SP									
SR				1					
LT				0.976	0.861–1.106	0.700	0.837	0.720–0.973	0.020^a^
Pathology									
AD	1								
SCC	1.324	1.019–1.720	0.036^a^	1.173	1.051–1.308	<0.001^a^	1.230	1.097–1.379	<0.001^a^

HR, hazard ratio; UD, undifferentiated; LN, lymph node; AD, adenocarcinoma SCC, squamous cell carcinoma; SR, sublobar resection; LR, lobectomy; SP, surgical procedure.

^a^ Difference was statistically significant.

### Subgroup analyses of the population stratified by surgical procedures

To further investigate the effects of the surgical procedures on OS and LCSS in patients, the full cohort was stratified by surgical procedures. As shown in Tables 6 and 7, no matter what surgical procedures were performed, LSQCC was associated with worse OS and LCSS compared with LUAD. Furthermore, age, gender, tumor differentiation grade, tumor size, and the number of resected lymph nodes were independent prognostic factors for OS and LCSS (Tables 6 and 7).

**Table 6:** Multivariate regression analyses for overall survival stratified by surgical procedure.

	Sublobar resection			Lobectomy		
	HR	95% CI	*P*	HR	95% CI	*P*
Age						
≤69	1			1		
>69	1.536	1.439–1.640	<0.001^a^	1.669	1.596–1.745	<0.001^a^
Gender						
Male	1			1		
Female	0.742	0.697–0.790	<0.001^a^	0.73	0.698–0.763	<0.001^a^
Grade						
Well/moderate	1			1		
Poor/UD	1.288	1.198–1.384	<0.001^a^	1.24	1.181–1.301	<0.001^a^
Unknown	1.052	0.938–1.179	0.385	0.954	0.868–1.049	0.331
Resected LNs						
0	1			1		
1–3	0.778	0.721–0.840	<0.001^a^	0.71	0.633–0.795	<0.001^a^
≥4	0.627	0.578–0.680	<0.001^a^	0.573	0.516–0.638	<0.001^a^
Unknown	0.746	0.639–0.872	<0.001^a^	0.651	0.567–0.747	<0.001^a^
Tumor size (mm)						
≤10	1			1		
11–20	1.16	1.070–1.259	<0.001^a^	1.065	0.981–1.156	0.135
21–30	1.374	1.248–1.512	<0.001^a^	1.268	1.166–1.378	<0.001^a^
Pathology						
AD	1					
SCC	1.353	1.263–1.449	<0.001^a^	1.429	1.360–1.502	<0.001^a^

HR, hazard ratio; UD, undifferentiated; LN, lymph node; AD, adenocarcinoma; SCC, squamous cell carcinoma.

^a^ Difference was statistically significant.

**Table 7:** Multivariate regression analyses for lung cancer–specific survival stratified by surgical procedures.

	Sublobar resection			Lobectomy		
	HR	95% CI	*P*	HR	95% CI	*P*
Age						
≤69	1			1		
>69	1.156	1.015–1.318	0.029^a^	1.155	1.068–1.248	<0.001^a^
Gender						
Male	1			1		
Female	0.836	0.735–0.951	0.006^a^	0.818	0.757–0.884	<0.001^a^
Grade						
Well/moderate	1			1		
Poor/UD	1.482	1.283–1.711	<0.001^a^	1.489	1.365–1.625	<0.001^a^
Unknown	0.925	0.718–1.193	0.549	1.017	0.856–1.209	0.844
Resected LNs						
0	1			1		
1–3	0.828	0.708–0.969	0.018^a^	0.81	0.655–1.002	0.052
≥4	0.718	0.611–0.844	<0.001^a^	0.65	0.538–0.784	<0.001^a^
Unknown	0.655	0.464–0.924	0.016^a^	0.763	0.602–0.968	0.026^a^
Tumor size (mm)						
≤10	1			1		
11–20	1.534	1.273–1.850	<0.001^a^	1.316	1.123–1.541	0.001^a^
21–30	2.336	1.901–2.870	<0.001^a^	1.733	1.478–2.033	<0.001^a^
Pathology						
AD	1			1		
SCC	1.2	1.040–1.385	0.012^a^	1.204	1.101–1.316	<0.001^a^

HR, hazard ratio; UD, undifferentiated; LN, lymph node; AD, adenocarcinoma; SCC, squamous cell carcinoma.

^a^ Difference was statistically significant.

## Discussion

In this study, we attempted to explore the impact of the histological types on the prognosis of IA NSCLC patients. We found that IA NSCLC patients with squamous cell carcinoma were at a significantly greater risk of lower survival compared with those with adenocarcinoma. After PSM analysis, squamous cell carcinoma was still closely associated with shorter OS and LCSS. To further explore the effect of histology type on OS and LCSS, the full cohort of the study population was stratified by tumor size and surgical procedures for further analysis.

Although many studies have been conducted on the relationship between histology and prognosis in patients with IA NSCLC, other variables were mainly explored, and the role of histological types in prognosis was seldom comprehensively analyzed. However, the survival rate of LSQCC is different from that of LUAD in the same TNM stage. Strand et al. (4) revealed that LSQCC was associated with favorable survival rate compared with LUAD, which was also supported by Wisnivesky's study (3). Some other studies pointed out that LUAD yielded better survival rate than LSQCC (5–7). However, some researchers found that there was no significant difference in survival rates between LSQCC and LUAD (17, 18). Our study was based on a large population and focused on the impact of histological type on the prognosis of IA-stage LSQCC and LUAD patients, and we demonstrated that LSQCC was associated with worse OS and LCSS. Moreover, subgroup analyses stratified by tumor size demonstrated that adenocarcinoma was closely related to better OS and LCSS compared with squamous cell carcinoma regardless of tumor size. When the cohort was stratified by surgical procedures, the results showed that, no matter what surgical procedures were performed, adenocarcinoma was associated with better OS and LCSS compared with squamous cell carcinoma. Most LSQCC patients had a history of smoking and therefore were likely to have a higher incidence of comorbidities such as chronic obstructive pulmonary disease and heart disease compared with non-squamous NSCLC (19, 20). Also, LSQCC is likely to be centrally located, and as a result, it has a higher probability of invading blood vessels (21). Thus, we speculate that the above-mentioned evidence might account for why LSQCC was associated with worse OS and LCSS.

Recently, some studies pointed out that the adenocarcinoma and squamous cell carcinoma are significantly different in many prognostic factors such as age, tumor location, smoking status, gender, pathological stage, clinical TNM stage, tumor differentiation grade, and survival (5, 6). Our study also revealed that older age, male, poor tumor differentiation, a larger number of resected lymph nodes, and larger tumor size were associated with poor OS and LCSS.

Certainly, our study has some limitations. Recently, great progress has been made in targeted therapies and immunotherapies. These patients who received these therapies may survive for a longer period than those who did not. However, IA-stage NSCLC patients were less likely to receive such treatments; thus, there is little possibility that our study has been substantially affected. Second, as a result of the nature of the retrospective study, some bias was inevitable. Our study performed multivariate Cox regression analyses to remove potential bias as much as possible. Third, information about the R status of patients who received surgery was not provided, which was closely associated with tumor recurrence and outcome. However, the early-stage tumor was most likely to be completely resected, thus achieving R0 status. Finally, we did not have any information on comorbidities, functional status, or pulmonary function of the study population, and we will explore the relationship between these factors and survival in our independent cohort. Moreover, our results need to be further validated in a large randomized cohort study in the future.

Taken together, our study demonstrated that LSQCC predicted worse OS and LCSS in IA-stage NSCLC patients compared with LUAD. The outcomes of LSQCC and LUAD were quite different, and we feel that the two histologic types need to be analyzed differently.

## Data Availability

The datasets presented in this study can be found in online repositories. The names of the repository/repositories and accession number(s) can be found in the article/Supplementary Material.
