# Supplementary figures and images for: Squamous cell carcinoma predicts worse prognosis than adenocarcinoma in stage IA lung cancer patients: A population-based propensity score matching analysis
Source: Front Surg. 2022 Aug 23;9:944032. doi: 10.3389/fsurg.2022.944032 (PMC9461700; doi:10.3389/fsurg.2022.944032)

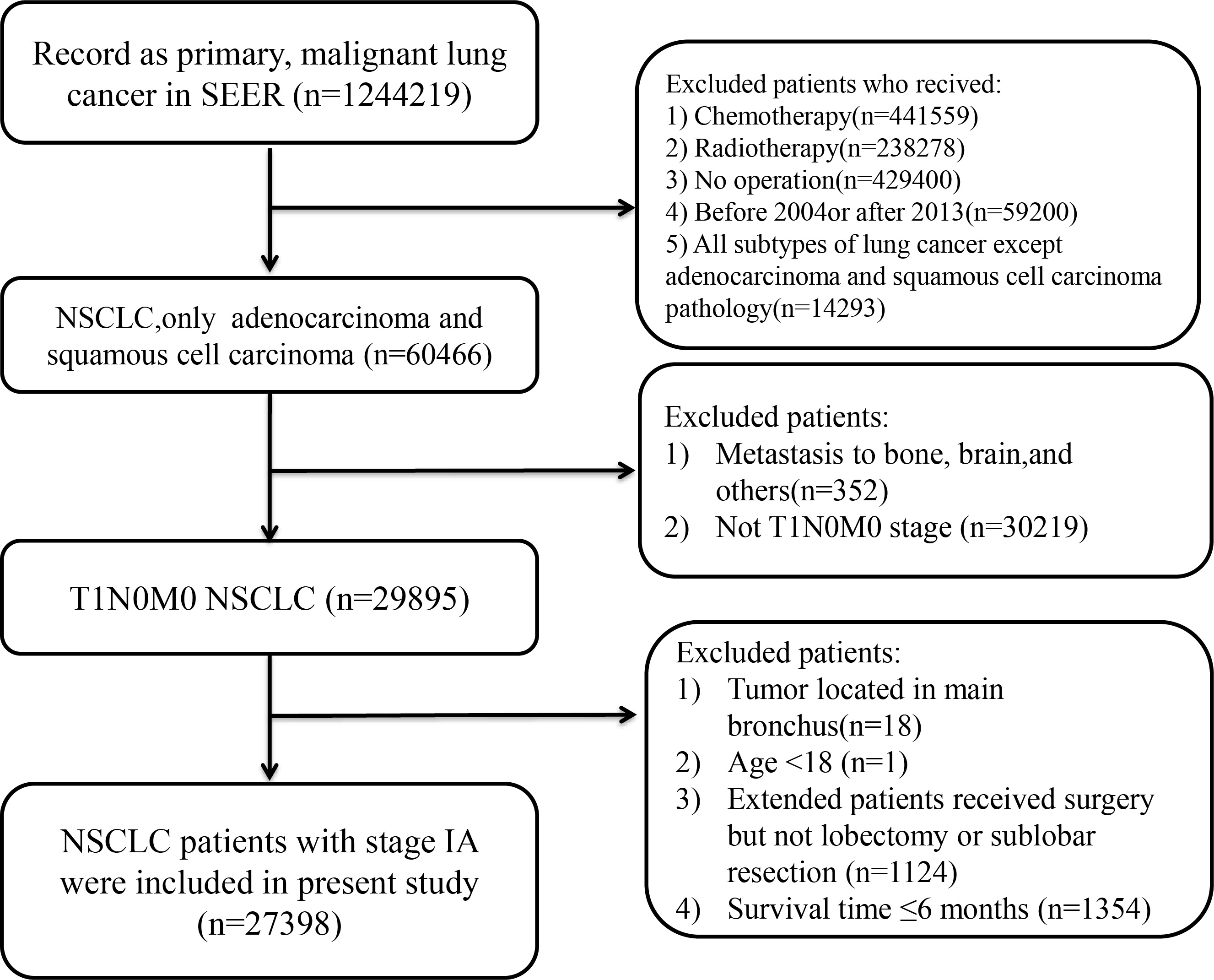

Supplement: Supplementary file 1 [file Image_1_v1.jpeg]

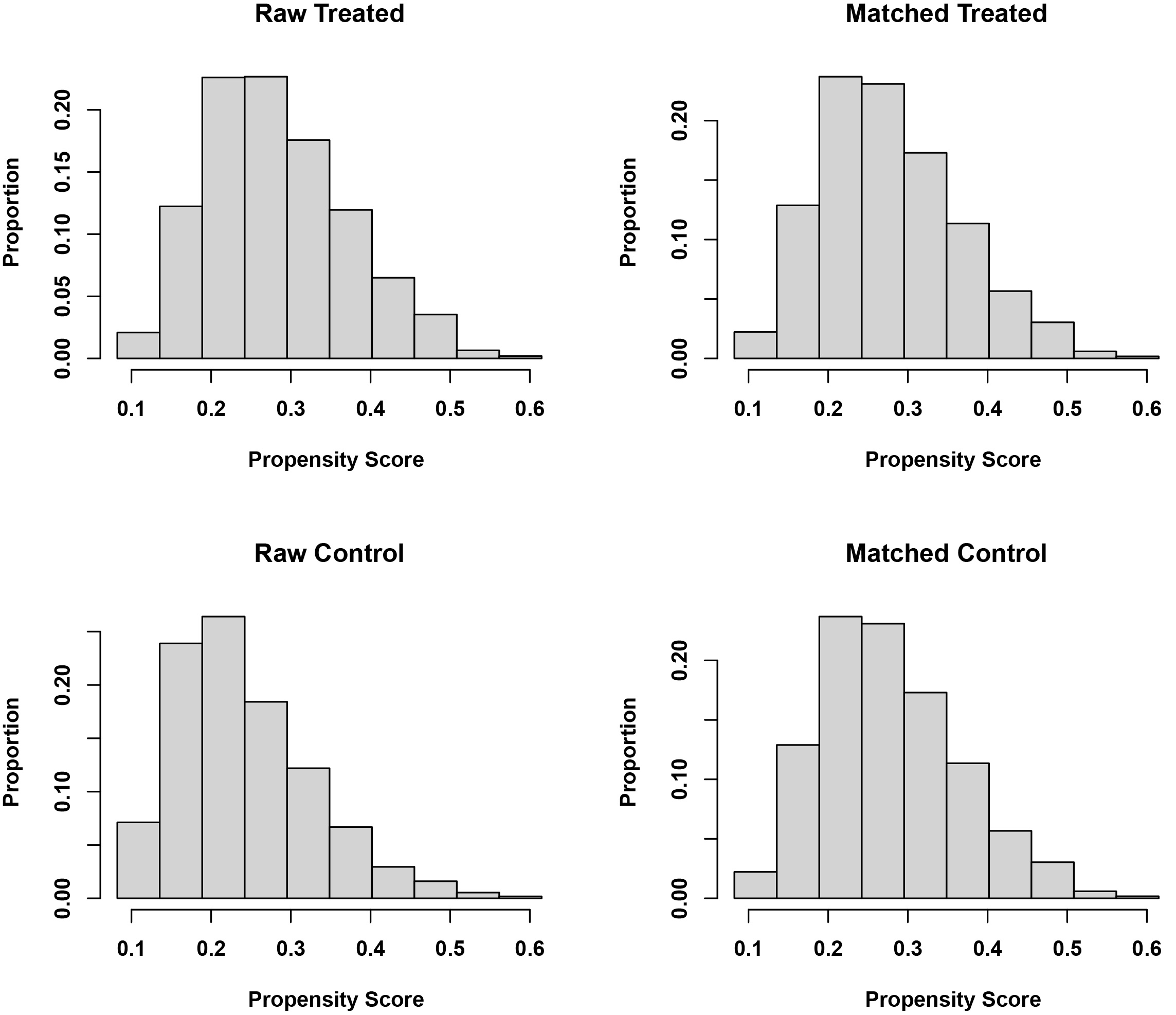

Supplement: Supplementary file 2 [file Image_2_v1.jpeg]
